# Advances in Lignocellulose-Degrading Enzyme Discovery from Anaerobic Rumen Fungi

**DOI:** 10.3390/microorganisms13122826

**Published:** 2025-12-12

**Authors:** Rajan Dhakal, Wei Guo, Ricardo Augusto M. Vieira, Leluo Guan, André Luis Alves Neves

**Affiliations:** 1 Department of Veterinary and Animal Sciences, Faculty of Health and Medical Sciences, University of Copenhagen, Grønnegårdsvej 3, DK-1870 Frederiksberg C, Denmark; dhakal@sund.ku.dk; 2 Key Laboratory of Plateau Mountain Animal Genetics, Breeding and Reproduction, Ministry of Education, College of Animal Science, Guizhou University, Guiyang 550025, China; guowei6866@gmail.com; 3 Laboratório de Zootecnia, Universidade Estadual do Norte Fluminense, Avenida Alberto Lamego 2000, Campos dos Goytacazes 28013-602, RJ, Brazil; ramvieira@uenf.br; 4 Faculty of Land and Food Systems, University of British Columbia, Vancouver, BC 2205, Canada

**Keywords:** enzyme discovery, lignocellulose bioconversion, biotechnology, metagenomics

## Abstract

Anaerobic fungi (phylum Neocallimastigomycota) play a crucial role in degrading forages and fibrous foods in the gastrointestinal tract of mammalian herbivores, particularly ruminants. Currently, they are classified into twenty-two genera; however, recent research suggests the occurrence of several novel taxa that require further characterization. Anaerobic rumen fungi play a pivotal role in lignocellulose degradation due to their unique enzymatic capabilities. This review explores the enzymatic systems of rumen anaerobic fungi, highlighting their ability to produce a diverse array of carbohydrate-active enzymes (CAZymes), such as cellulases, hemicellulases, and pectinases. These enzymes facilitate the breakdown of complex plant polymers, making anaerobic fungi essential contributors to fiber degradation in the rumen ecosystem and valuable resources for biotechnological applications. This review summarizes the structural and functional diversity of fungal CAZymes, and the mechanical disruption of plant cell walls by fungal rhizoidal networks is discussed, showcasing the ability of fungi to enhance substrate accessibility and facilitate microbial colonization. Recent studies using genomic, transcriptomic, and biochemical approaches have uncovered several novel CAZymes in anaerobic fungi, including multifunctional xylanases, β-glucosidases, and esterases. These findings highlight the continued expansion of fungal enzyme repertoires and their potential for biotechnology and feed applications. Continued research in this field will enhance our understanding of microbial ecology and enzyme function, paving the way for applications that address global challenges in energy, food security, and environmental sustainability.

## 1. Introduction

**Figure 1:**
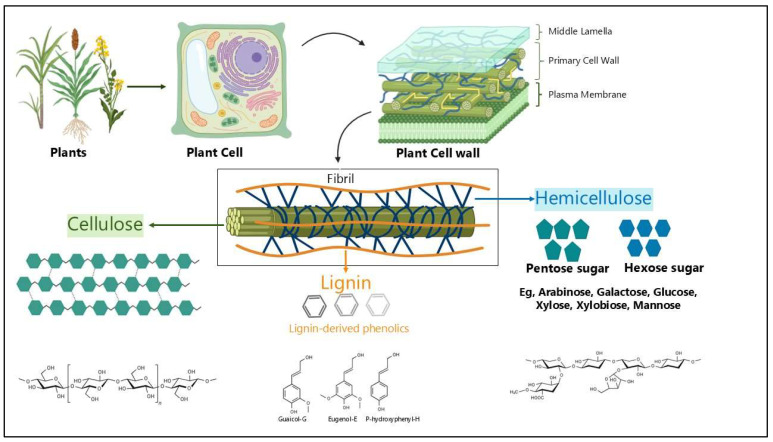
Schematic representations of plant cell walls and their main components: cellulose, hemicellulose, and lignin. Adapted from Wunderlich et al. [2], published under Creative Commons Attribution 4.0 International (CC BY 4.0): https://link.springer.com/article/10.1186/s42523-022-00224-6 (accessed on 3 December 2025). Created in BioRender by Dhakal, R. (2025) [3]: https://BioRender.com/wr521rv.

A major bottleneck in converting biomass into ruminant feed sources and biofuels is the lack of an efficient enzymatic system for deconstructing the plant cell wall matrix and releasing all the fermentable sugars. The plant cell wall matrix is the major structural component of plants and consists of three main polymers (Figure 1): cellulose, hemicellulose, and lignin [1].

Cellulose is a linear biopolymer of anhydroglucopyranose molecules that are connected by β-1,4-glycosidic bonds. Adjacent cellulose chains are bound together by hydrogen bonds, hydrophobic interactions, and Van der Waals forces, which cause a parallel alignment of crystalline structures known as microfibrils [4]. The second major component of lignocellulose is hemicellulose, which consists of heterogeneous polymer of pentoses (including xylose and arabinose), hexoses (mainly mannose, glucose, and galactose) and sugar acids [5]. The composition of hemicelluloses in nature varies considerably depending on plant material [6,7]. Lignin is the third primary polymer in the lignocellulosic biomass. Lignin is a complex, cross-linked phenolic polymer synthesized from three primary monolignols (p-coumaryl, coniferyl, and sinapyl alcohol) [1].

Those three major components of the plant cell wall (lignin, cellulose, and hemicellulose) form cross-link structures, which are the primary barriers to carbohydrase enzymes gaining access to specific substrates within the lignocellulosic fiber. Among these components, lignin is the most recalcitrant, posing significant challenges to rumen microorganisms during degradation, as its complex cross-linking with hemicellulose limits substrate accessibility and hinders the overall digestibility of the plant cell wall matrix [8,9,10]. However, lignin digestion is not only impaired by the limited fibrolytic capacity of the microbiome but also by the surface area available for enzyme attachment and by microbial accessibility to the inner parts of fibrous plant tissues [10,11]. Thus, lignin solubilization may represent a crucial step in enhancing the digestibility of fibrous biomass through microbial fermentation processes.

## 2. Anaerobic Fungi and Their Role in the Degradation of Recalcitrant Plant Cell Walls

Orpin [12,13] was the first to describe the life cycle of anaerobic fungi in 1975, identifying their presence in the gastrointestinal tract of herbivores, particularly in the rumen and caecum. These strictly anaerobic microbes, belonging to the phylum Neocallimastigomycota, play a key role in the degradation of lignocellulosic feedstuffs within the gut of ruminant and non-ruminant herbivores. Anaerobic fungi are currently grouped into twenty-two genera, based on recent taxonomic revisions [14]. They were characterized primarily using molecular biology and classical microscopy techniques [15,16], and have long been studied using traditional approaches. However, the advent of next-generation sequencing has uncovered a far greater biodiversity within these fungi than previously recognized [17], highlighting the limitations of earlier classification methods and opening new avenues for understanding their ecological roles. Each genus is distinguishable by morphological features, such as thallus morphology (monocentric vs. polycentric), rhizoid morphology (filamentous vs. bulbous), and zoospore flagellation (monoflagellate vs. polyflagellate) [18,19] (Table 1).

**Figure 2:**
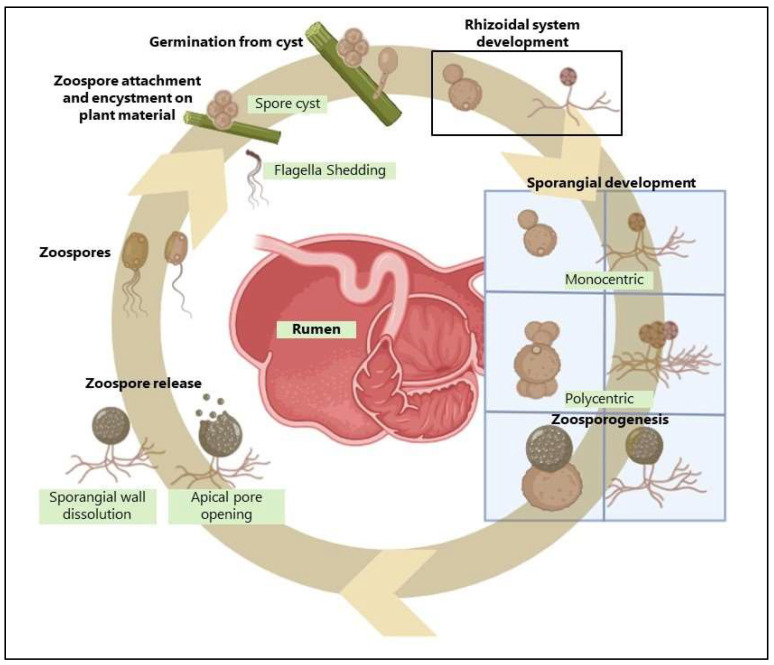
The life cycle of Neocallimastigomycota in the rumen. Created in BioRender, by Dhakal, R. (2025) [22]: https://BioRender.com/38ghp37.

Anaerobic fungi have a complex lifecycle consisting of a free-living zoospore stage and an attached phase characterized by mycelial growth and sporangia [13,20]. The life cycle of Neocallimastigomycota (Figure 2), a phylum of anaerobic fungi, primarily involves asexual reproduction through the production of motile zoospores [21]. These zoospores are released from sporangia and use chemotaxis to locate and colonize plant material within the digestive tracts of herbivores. Upon reaching the plant cell surface, the zoospores germinate, forming a rhizoidal network that penetrates and physically disrupts the plant tissue. This network eventually develops into a new sporangium, completing the life cycle.

**Table 1:** Known genera of anaerobic fungi, adapted from Ho et al. and McAllister et al. [16,23].

Genus	Flagella per Zoospore	Thallus	Rhizoids
*Agriosomyces*	Uniflagellate	Monocentric	Filamentous
*Aklioshbomyces*	Uniflagellate	Monocentric	Filamentous
*Anaeromyces*	Uniflagellate	Polycentric	Filamentous
*Aestipascuomyces*	Polyflagellated	Monocentric	Filamentous
*Astrotestudinimyces*	Monoflagellated	Polycentri	Filamentous
*Buwchfawromyces*	Uniflagellate	Monocentric	Filamentous
*Caecomyces*	Uniflagellate	Monocentric	Bulbous
*Capellomyces*	Uniflagellate	Monocentric	Filamentous
*Cyllamyces*	Uniflagellate	Polycentric	Bulbous
*Feramyces*	Polyflagellate	Monocentric	Filamentous
*Ghazallomyces*	Polyflagellate	Monocentric	Filamentous
*Joblinomyces*	Uniflagellate	Monocentric	Filamentous
*Khoyollomyces*	Uniflagellate	Monocentric	Filamentous
*Liebetanzomyces*	Uniflagellate	Monocentric	Filamentous
*Neocallimastix*	Polyflagellate	Monocentric	Filamentous
*Oontomyces*	Uniflagellate	Monocentric	Filamentous
*Orpinomyces*	Polyflagellate	Polycentric	Filamentous
*Pecoramyces*	Uniflagellate	Monocentric	Filamentous
*Piromyces*	Uniflagellate	Monocentric	Filamentous
*Testudinimyces*	Monoflagellated	Polycentric	Filamentous
*Paucimyces*	Monoflagellated	Polycentric	Filamentous
*Tahromyces*	Uniflagellate	Monocentric	Filamentous

**Figure 3:**
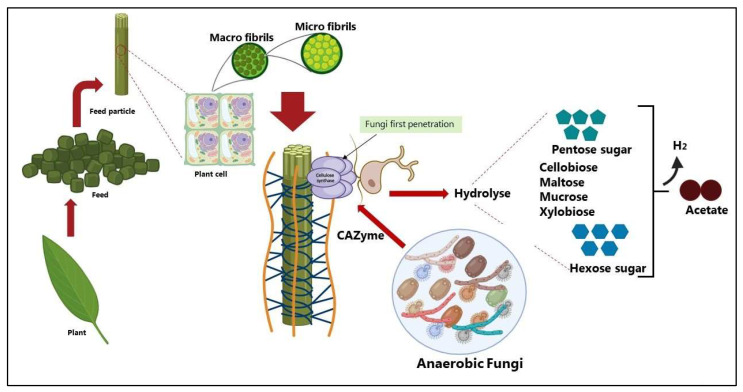
Schematic representations of fiber degradation by anaerobic fungi in ruminants. Created in BioRender by Dhakal, R. (2025) [35]: https://BioRender.com/kb1qr0p.

During this attached growth phase, they act in concert with fibrolytic bacteria to break down plant materials and form symbiotic relationships with methanogenic archaea [24], both of which enhance fiber degradation. However, unlike bacteria, fungi can physically penetrate and disrupt the plant cell wall (Figure 2 and Figure 3) using an appressorium-like structure [25,26]. This disruption may increase the surface area for enzyme activity and bacterial attachment [13,25,26], consequently enhancing degradation of plant cell walls [27].

Several experiments have provided insights into the importance of anaerobic fungi in plant cell wall disruption and their contribution to fiber digestion, feed intake, rumen fermentation, and overall rumen metabolism [28]. The removal of these fungi from the rumen results in reduced voluntary feed intake and dry matter degradation, indicating their essential role in feed digestion [29,30,31]. In general, the elimination of anaerobic fungi from the rumen significantly reduces the degradation of dry matter, neutral detergent fiber, acid detergent fiber, and carboxymethylcellulase activity [32,33,34].

## 3. Anaerobic Fungi and Their CAZymes

Microbes play a significant role in regulating the biochemical processes involved in feed digestion within the rumen [1,36] and serve as valuable sources of enzymes with various biotechnological applications [4]. Growing interest in the enzymatic saccharification of the plant cell wall matrix has led to the creation of an extensive database cataloging CAZymes families—structurally related enzymes, as well as carbohydrate-binding modules (CBMs). These databases are continually updated to cover all known CAZymes across various organisms and areas of glycoscience. CAZymes and their associated CBMs are classified based on sequence similarity and encompass biocatalysts that modify and cleave carbohydrates (glycoside hydrolases—GHs, polysaccharide lyases—PLs, and carbohydrate esterases—CEs), as well as synthesize them (glycosyltransferases) [37,38]. As of 2025, the CAZy database reports nearly 200 glycoside hydrolase (GH) families and over 100 carbohydrate-binding module (CBM) families, reflecting the rapidly expanding diversity of carbohydrate-active enzymes (https://www.cazy.org/, accessed on 5 December 2025).

More recent taxonomic updates indicate that Neocallimastigomycota comprise 22 genera (Table 1) and 38 species [14,23]. Fungi can degrade recalcitrant plant cell wall materials because of their ability to secrete a broad range of hydrolytic CAZymes [39,40,41]. Some of these enzymes are free, but most are bound to cellulosome complexes and exhibit unique characteristics compared to those from other microbes, such as fibrolytic bacteria [42,43]. Genome sequencing of anaerobic rumen fungus *Orpinomyces* strain C1A revealed a broader enzyme repertoire than that of aerobic fungi (*Dikarya*), consisting of 357 glycosyl hydrolases, 92 carbohydrate esterases, and 24 pectate lyases. Horizontal gene transfer from bacteria may help explain why anaerobic fungi have evolved robust and impressive cellulolytic and hemicellulolytic capability [15]. Among the diverse CAZymes secreted by anaerobic fungi, many are organized into large extracellular multi-enzyme complexes known as cellulosomes. These structures play a pivotal role in mediating plant fiber degradation and represent a unique adaptation among fibrolytic microbes [44].

### 3.1. Cellulosomes in Anaerobic Fungi

Cellulosomes are multiprotein complexes and can be categorized based on their structural and functional components [45]. These multienzyme complexes are primarily composed of two types of subunits: scaffoldins and catalytic subunits [46]. Each of these subunits contains various covalently linked modules further classified into four categories: assembly modules, catalytic modules, substrate-binding modules and cell-binding modules. Anaerobic fungi of the Neocallimastigomycota phylum possess powerful cellulosomes for plant biomass degradation [45]. These fungal cellulosomes are evolutionarily distinct from bacterial counterparts, featuring unique scaffoldin and dockerin domains [45,47]. Genomic analyses reveal an abundance of carbohydrate-active enzymes and cellulosome-related genes in these fungi, highlighting their exceptional cellulolytic capabilities [48]. The cellulosome structure can be exploited to design chimeric enzymes with enhanced properties, such as thermostability [47]. Cellulosome production and localization patterns vary across the fungal life cycle and environmental conditions, influencing biomass degradation efficiency [49]. Understanding these mechanisms is crucial for developing anaerobic fungi as platforms for converting waste biomass into valuable products, such as biofuels and chemicals [48,49].

Unlike bacterial cellulosomes, fungal cellulosomes contain long tandem arrays of fungal-specific dockerin repeats and unusually large multi-catalytic enzymes, often combining GH, CE, and CBMs within a single polypeptide [45,47]. Fungal scaffoldins and dockerins are sequence-divergent from their bacterial counterparts and do not resemble the canonical cohesin–dockerin architecture of Clostridium spp., indicating that fungal cellulosomes represent an independently evolved strategy for lignocellulose deconstruction [45,46,47,49].

### 3.2. Cellulases

Cellulases are family members of the broad group of GHs (e.g., GH1, GH3, GH5, GH6, GH7, GH8, GH9, GH45, GH48, GH124), which have gained interest for a number of biotechnological applications. This group of enzymes synergistically hydrolyze the β-1,4-glucosidic bonds in cellulose through three different types of enzymes [13]. Based on the structure and functionality, cellulases have been classified into three groups: (1) Endoglucanases, a group of cellulases that exhibits a deep cleft or groove to accommodate the cellulose chain at any point along its length in order to cleave internal bonds at amorphous sites of new chain ends; (2) Exoglucanases, in contrast, are a group of cellulases that possess the active site in an extended loop that forms a tunnel, through which one of the termini of a cellulose chain can be threaded; and (3) β-glucosidases, which are cellulases that hydrolyze cellobiose to generate two molecules of glucose, and are often associated with the microbial cell surface when cellodextrins are transported into the cell [8]. All three major cellulase types have been reported in the phylum Neocallimastigomycota [39,50,51,52], confirming that anaerobic rumen fungi are reservoirs of highly efficient cellulases [15].

Compared to aerobic fungi, anaerobic fungal cellulases frequently occur as large, multi-domain CAZymes and are often incorporated into cellulosomes, whereas many cellulolytic bacteria rely on smaller, single-function enzymes [45,47,49,53,54].

### 3.3. Hemicellulases

In contrast to cellulose degradation, the digestion of hemicelluloses poses a different challenge, as hemicelluloses include widely different types of sugars or non-sugar constituents with different types of bonds. Hemicellulases can be divided into two main groups: (a) those that cleave the mainchain backbone (e.g., mannanases and xylanases), and (b) those that degrade sidechain substituents or short end products (e.g., arabinofuranosidase) [15,55]. Consequently, the catalytic modules of hemicellulases can be either glycosyl hydrolases that hydrolyze glycosidic bonds, or carbohydrate esterases, which hydrolyze ester linkages of acetate or ferulic acid side groups [13,15,55]. Due to the different structures of hemicellulose, several enzymes are required for their catabolism. To date, anaerobic fungi have been reported to provide all the enzymes required to degrade the major hemicellulose constituents of the plant cell wall, namely β-glucans, mannans, and xylans [15,56]. In some cases, xylanase activity is higher than cellulase activity [57].

Omics studies show that anaerobic fungi encode an exceptionally broad array of xylan-active GH families—such as GH10, GH11, GH43, GH51, and GH115—many of which occur in multi-catalytic proteins, unlike the simpler xylanase repertoires typically found in rumen bacteria and aerobic fungi [58,59,60,61,62].

#### 3.3.1. Mannanases

Mannan is a hemicellulose component consisting of β-1,4 linkages between mannose monomers that form the hemicellulose cross-linkages [17]. β-Mannanases (GH26) hydrolyze mannan-based hemicelluloses and release short β-1,4-manno-oligomers, which can be further hydrolyzed to mannose by the action of β-mannosidases. Recently, researchers have applied exogenous β-mannanases from *Aspergillus niger* in the animal feed industry as an additive to improve feed conversion efficiency in dairy cattle [18,63,64]. However, the rumen microbiome can degrade mannan, as demonstrated in the discovery of a multifunctional glycosyl hydrolase encoded in the genome of the bacterium *Prevotella bryantti B14* [19].

Mannanase-encoding genes, including GH5 and GH26 families, are widely represented in the genomes and transcriptomes of anaerobic fungi such as *Caecomyces, Neocallimastix,* and *Piromyces,* highlighting their direct role in mannan degradation [58,65,66].

#### 3.3.2. Arabinofuranosidases

Arabinose is found in conjunction with xylan as a hemicellulose component of plant cell walls, with arabinose units being attached to xylan via alpha-1,2,1,3,1,5 or linked to C2 or C3 positions on the arabinoxylan chain [13]. In the rumen, arabinose units can be cleaved off the xylose backbone by arabinofuranosidases (e.g., GH3, GH43) expressed by rumen bacteria such as *Ruminococcus albus* [28,67,68].

Anaerobic fungi also express arabinofuranosidases from GH43 and GH51 families, as demonstrated across multiple genera through genomic and transcriptomic surveys [58,65,66].

#### 3.3.3. Ferulic Acid Esterases

Ferulic acid esterase (e.g., CE1) is a group of enzymes that forms a subclass of carboxylic ester hydrolases. These enzymes hydrolyze the bonds between hydroxycinnamates and sugars to release ferulic acid [12,19,69,70]. In the rumen, these ester bonds are cleaved by ferulic acid esterases encoded in the genome of the rumen fungi *Anaeromyces mucronatus* [20,71].

Anaerobic fungi possess CE1 feruloyl esterases, including recently described multifunctional enzymes capable of cleaving both ferulate–sugar and xylan linkages, which play a key role in disrupting lignin–carbohydrate complexes [60,72].

#### 3.3.4. p-Coumaric Acid Esterases

p-Coumaric acid esterase or p-coumaroyl esterase (e.g., CE1) is an essential enzyme for efficient degradation of lignocellulose biomass in the rumen [14,18,19]. This enzyme targets the p-Coumaroyl ester bonds that connect lignin to hemicelluloses, releasing p-Coumaric acid. This type of enzyme is so far mainly reported from anaerobic fungi (Neocallimastigomycota); evidence in rumen bacteria is limited/underexplored [24], further strengthening the ecological role and significance of anaerobic fungi for deconstructing lignocellulose biomass in the rumen [18,25,26].

p-Coumaroyl esterases have been described primarily in anaerobic fungi, where they mediate cleavage of ester linkages between lignin and hemicellulose components, facilitating access for cellulases and xylanases [60,72].

#### 3.3.5. Xylanases

The chemical structure of xylan molecule is complex and consists of β-1,4 linked xylopyranosyl residues and contains sidechains with acetyl group and L-arabinofuranosyl residues. Rumen fungi produce xylanases (e.g., GH5, GH8, GH10, GH11, GH26, GH30, GH31, GH39, GH43, GH51, GH74, GH94, and GH115) which are responsible for the hydrolysis of xylan by breaking the glycosidic linkages present in the xylan backbone [73]. Like cellulases, xylan-degrading enzymes include endoxylanases and β-xylosidases, while related carbohydrate esterases such as acetyl xylan esterases (AXEs) act on acetyl side groups of xylan rather than on the xylan backbone itself [74]. Therefore, AXEs complement xylanase activity but are not considered true xylanases. All three enzymes hydrolyze the xylan molecule, rendering D-xylose sugar [75]. The growing interest in xylanases is evidenced by the vast number of research papers published in recent years describing numerous xylanases applications in the pulp and paper industries [74,76], and as exogenous enzyme preparations marketed by the animal feed industry [41].

Recent studies confirm that anaerobic fungi produce a diverse suite of xylanases spanning GH10, GH11, GH43, and GH115 families, including newly characterized enzymes with broad specificity and industrial potential [58,59,61,62].

### 3.4. Pectinases

Pectin exists in the primary cell wall and represents the plant’s first line of defense against dehydration and penetration by phytopathogens. Pectin’s structure is a backbone of alpha-1,4-linked residues of D-galacturonate that is degraded by rumen pectinolytic enzymes (e.g., PL11, GH28), including pectin lyases, polygalacturonases, and pectin methylesterases [70]. One of the major pectinolytic bacterial species that reside in the rumen is *Lachnospira multiparus*, which produces pectin lyases and pectin methylesterases [77,78]. In addition to that bacterial species, rumen fungi also exhibit pectinolytic enzymes [12]. Early studies demonstrated the production of polygalacturonases and pectin methylesterases by anaerobic rumen fungi [79], and recent genomic analyses continue to identify CAZyme families (e.g., GH28, PL11) associated with pectin degradation in Neocallimastigomycota [58,66].

### 3.5. Polyphenol and Lignin-Degrading Enzyme*s*

Feed consumed by ruminants contains not only the nutrients required by the host for maintenance and production but also holds naturally occurring plant secondary compounds such as tannins, saponins, phenolic acids, and silica that usually cause adverse effects on the activity of fibrolytic enzymes [80,81]. However, some gastrointestinal microbes of ruminants are able to break down tannin-protein complexes through an enzyme known as tannin acyl hydrolase (tannase), which catalyzes the hydrolysis of ester bonds present in gallotannins, complex tannins, and gallic acid esters [48,82,83,84]. While anaerobic fungi of the Neocallimastigomycota phylum are pivotal in the breakdown of fibrous plant components, their direct involvement in polyphenol degradation is limited due to the absence of specific lignin-degrading enzymes. Genomic analyses have revealed that these fungi lack genes encoding for lignin-degrading auxiliary activity enzymes, including laccases and peroxidases. This absence suggests a reduced intrinsic ability to decompose lignin and other polyphenolic substances [48]. In contrast to aerobic fungi, anaerobic fungi lack the enzymatic machinery to catabolize lignin. The enzymatic reaction to cleave the aromatic ring requires oxygen and, therefore, cannot take place in an anaerobic environment. However, it has been shown that *Neocallimastix* sp. could mediate the degradation of up to 34% of plant biomass-associated lignin by a physical alteration or chemical modification of the lignin structure instead of enzymatic catabolism [50].

Recent research has further advanced our understanding of lignin modification by anaerobic fungi. Lankiewicz et al. [72] provided evidence that certain Neocallimastigomycota harbor auxiliary enzyme families with potential oxidative or reductive activity toward lignin-derived aromatics, challenging the traditional view that lignin degradation is limited to aerobic fungi. These findings suggest that anaerobic fungal metabolism may contribute to lignin transformation through non-oxidative or redox-assisted mechanisms under anaerobic conditions, broadening the scope of their ecological function and biotechnological potential.

### 3.6. Recent Advances in Lignocellulose-Degrading Enzyme Discovery from Anaerobic Fungi

In recent years, significant progress has been made in researching the diversity, structure, and function of lignocellulose-degrading enzymes from anaerobic fungi. A large-scale screening campaign of putative carbohydrate-active enzymes identified a novel xylanase with broad substrate specificity and high catalytic efficiency from anaerobic gut fungi [59]. Similarly, genomic and transcriptomic characterization of *Neocallimastix cameroonii* var. *constans* revealed an expanded repertoire of CAZymes, providing new insights into the genomic basis of lignocellulose degradation in anaerobic fungi [58]. Structural and biochemical analyses have also advanced, with the discovery of multifunctional GH5 endoglucanases from *Piromyces finnis* [54] and detailed crystal structures of CelD (GH family 5 subfamily 4) from the same species, elucidating catalytic mechanisms and domain organization [53].

New β-glucosidases have been described, including a bifunctional enzyme from *Neocallimastix patriciarum* exhibiting both β-glucosidase and β-glucanase activity. In addition, *Pecoramyces ruminantium* F1 was shown to express a dual-functional feruloyl esterase–xylanase capable of cleaving both ester and glycosidic linkages, revealing enzymatic multifunctionality that enhances hemicellulose breakdown [60]. Complementary to these discoveries, studies of anaerobic gut fungi isolated from wild ruminants, such as *Neocallimastix* from Anatolian wild goat, continue to expand our taxonomic and enzymatic knowledge base, including novel xylanases, cellulases, and lichenases with potential for biotechnology applications [61].These findings, together with previous omics and structural studies [62,65,66], highlight continuing progress in fungal enzyme discovery, reinforcing the ecological and biotechnological significance of anaerobic fungi as a source of lignocellulose-degrading enzymes.

## 4. Approaches to Identifying Lignocellulolytic Enzymes in Anaerobic Rumen Fungi

**Figure 4:**
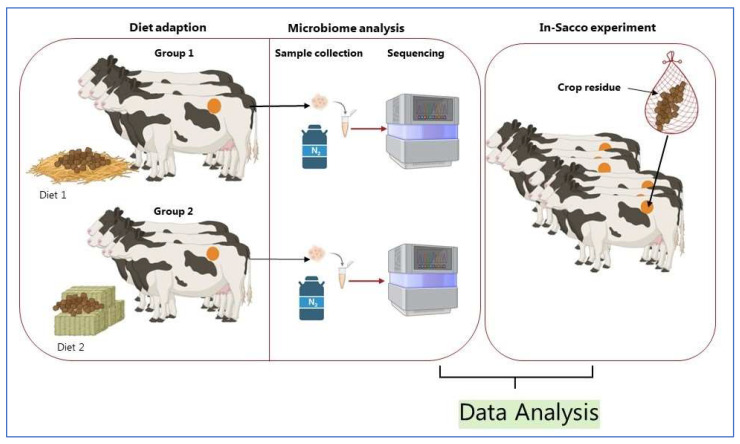
Schematic representation of assessing anaerobic fungi in ruminants. Created in BioRender by Dhakal, R. (2025) [85]: https://BioRender.com/t5vtb87.

Understanding the unique role of anaerobic fungi in lignocellulose degradation requires advanced tools and techniques that integrate microbial community analysis, enzymatic profiling, and bioinformatics approaches (Figure 4). These methods allow for identifying fungal strains with superior fiber-degrading capabilities and their subsequent application in biotechnological processes.

### 4.1. Microbial Community and CAZyme Analysis (General Approach)

Rumen samples need to be collected from animals adapted to high-fiber diets to investigate anaerobic fungi and ensure microbial stability (Figure 4). Cannulated cattle are commonly used, with samples obtained after a 14 to 28-day adaptation period [86]. Though there is no universal time for sampling, and rumen microbes are similar day to day [71], daily sampling over five days provides a robust dataset to monitor microbial changes [87,88]. Proper sample handling, including freezing at −80 °C and grinding under liquid nitrogen, ensures the integrity of DNA and RNA for metagenomic and metatranscriptomic analyses [89]. Molecular techniques, such as sequencing the Internal Transcribed Spacer 1 (ITS1) region, are employed to identify fungal taxa and assess diversity [90].

### 4.2. Evaluating Fiber Degradation Through In-Sacco Experiments

The in-sacco technique is a standard method to evaluate fiber degradation by anaerobic fungi. Fiber degradation in in sacco experiments depends on feed material, particle size, bag type, and ruminal conditions [91,92]. Polyester bags containing crop residues are incubated in the rumen for 1 to 144 h to assess the rate and extent of dry matter disappearance and fiber digestion. Rapid bacterial colonization is noted within 10 min, yet fiber disappearance follows a steady, linear path [93,94]. This method provides key kinetic parameters, including lag time and neutral detergent fiber digestibility. Such experiments are critical for correlating microbial activity with lignocellulosic degradation.

### 4.3. Isolation of Superior Anaerobic Fungal Strains

Anaerobic fungi isolated from animals with superior fiber-degrading capabilities are cultured on straw biomass using a basal growth medium to induce fibrolytic enzyme expression [95,96]. Biomass quantification and gas production measurements monitor strain growth over several days. This process identifies fungal strains with high enzymatic activity, enabling their use in enzyme discovery and biotechnological applications.

### 4.4. Enzyme Mining Tools

Advanced bioinformatics and omics-driven strategies are now central to discovering lignocellulose-degrading enzymes in anaerobic fungi. High-quality metagenomic and transcriptomic data enable genome annotation and comparative analyses that identify candidate CAZymes involved in plant-fiber deconstruction. Tools such as CAZy and dbCAN, combined with machine-learning prediction and docking simulations, have accelerated functional annotation and domain prediction in newly sequenced isolates [97,98,99].

Recent enzyme discovery pipelines integrate sequence-based screening with expression and biochemical validation. For instance, in silico mining of metatranscriptomes has revealed numerous previously uncharacterized dockerin-containing CAZymes, while recombinant expression systems now permit heterologous testing of fungal genes under anaerobic-like conditions [72,98]. Structure-guided engineering approaches are increasingly used to model thermostability and substrate specificity of CAZymes identified through these pipelines, bridging the gap between computational prediction and practical enzyme design.

These advances highlight how integrated omics, bioinformatics, and synthetic-biology tools are redefining enzyme mining in anaerobic fungi. The combination of functional genomics, structural modeling, and high-throughput screening is expected to accelerate the identification of novel lignocellulolytic enzymes with industrial potential.

## 5. Conclusions and Future Perspectives

Research on anaerobic fungi Neocallimastigomycota has highlighted their significant potential in the degradation of lignocellulosic biomass. These microbes possess unique enzymatic capabilities that enable them to break down complex plant polymers, which are otherwise resilient to degradation by most other microorganisms. The ability of anaerobic fungi to physically disrupt plant cell walls through the formation of rhizoidal networks provides a mechanical advantage in accessing and degrading plant biomass. The biotechnological potential of anaerobic fungi is vast. Their enzymes can be harnessed for various industrial applications, including the production of biofuels, animal feed, and other value-added products. Future research on anaerobic rumen fungi and their enzymes holds bigger potential for advancing our understanding of fiber degradation and its applications. Several key areas justify further investigation:

Enzyme Engineering: Optimizing fungal enzymes through protein engineering and directed evolution can enhance their stability, activity, and substrate specificity. Engineering efforts should focus on improving the performance of these enzymes under industrial conditions, such as high temperatures and varying pH levels.

Synthetic Biology: The development of synthetic biology approaches to construct microbial consortia that mimic the natural rumen environment can enhance fiber degradation. Co-culturing anaerobic fungi with other fibrolytic microorganisms, such as bacteria and archaea, can create synergistic interactions that improve overall biomass conversion.

Bioprocess Optimization: Integrating fungal enzymes into bioprocesses for biofuel production, performance enhancement on animals fed forages and fibrous foods, and other applications requires optimizing fermentation conditions, enzyme loading, and substrate pretreatment. Pilot-scale and industrial-scale studies will be key in translating laboratory findings into practical applications.

Future research should prioritize resolving the complete CAZyme inventories of anaerobic fungal genera, clarifying the organization and regulation of fungal cellulosomes across life stages, and determining how multi-catalytic enzymes evolved uniquely in these obligate anaerobes. Key gaps include limited structural data for fungal CE and PL families, incomplete genomic coverage of recently described genera, and poor understanding of how anaerobic fungi interact syntrophically with rumen bacteria and archaea to coordinate lignocellulose degradation.

## Data Availability

No new data were created or analyzed in this study. Data sharing is not applicable to this article.
